# Supplementary material for: Tracking the Genomic Evolution of SARS-CoV-2 for 29 Months in South Korea
Source: Viruses. 2023 Mar 29;15(4):873. doi: 10.3390/v15040873 (PMC10142693; doi:10.3390/v15040873)
Supplement: Supplementary file 1 [file viruses-15-00873-s001.zip › viruses-2237147-supplementary.pdf]

Table S1. Nucleotide sequences of the primers used for the SARS-CoV-2 strains

Primer name	Sequence (5' to 3')
Fragment 1_70-F	ACAAAAGAAAATGACTCTAAAGAGGGTTT
Fragment 1_71-R	TGGAAAAGAAAGGTAAGAACAAGTCCT
Fragment 2_72-F	ACACGTGGTGTTTATTACCCTGAC
Fragment 2_73-R	CACCAGCTGTCCAACCTGAAGA
Fragment 3_74-F	ACATCACTAGGTTTCAAACTTTACTTGC
Fragment 3_75-R	ACCACCAACCTTAGAATCAAGATTGT
Fragment 4_76-F	AGGGCAAACTGGAAAGATTGCT
Fragment 4_77-R	CAGCCCCTATTAAACAGCCTGC
Fragment 5_78-F	CAACTTACTCCTACTTGGCGTGT
Fragment 5_79-R	CATTTCATCTGTGAGCAAAGGTGG
Fragment 6_80-F	TTGCCTTGGTGATATTGCTGCT
Fragment 6_81-R	GTGAAGTTCTTTTCTTGTGCAGGG
Fragment 7_82-F	GGGCTATCATCTTATGTCCTTCCCT
Fragment 7_83-R	TTTGACTCCTTTGAGCACTGGC
Fragment 8_84-F	TGCTGTAGTTGTCTCAAGGGCT
Fragment 8_85-R	ACACAGTCTTTTACTCCAGATTCCC
Fragment 1-1_69-F	TGTCGCAAAATATACTCAACTGTGTCA
Fragment 1-1_70-R	TGACCTTCTTTTAAAGACATAACAGCAG
Fragment 1-2_71-F	ACAAATCCAATTCAGTTGTCTTCCTATTC
Fragment 1-2_72-R	ACTCTGAACTCACTTTCCATCCAAC
Fragment 3-1_73-F	CAATTTTGTAATGATCCATTTTTGGGTGT
Fragment 3-1_74-R	GCAACACAGTTGCTGATTCTCTTC
Fragment 3-2_75-F	AGAGTCCAACCAACAGAATCTATTGT
Fragment 3-2_76-R	ACACCTGTGCCTGTTAAACCAT

Table S2. Representative sequences for the Wild-type, Alpha, Epsilon, Delta, and Omicron (BA.1, BA.2, and BA.2.12.1) variants

Type	Representative strain	GenBank Accession number
Wild	SARS-CoV-2 isolate Wuhan-Hu-1, complete genome	NC045512
Alpha	SARS-CoV-2 isolate SARS-CoV-2/human/USA/FL-CDC-STM-P012/2020, complete genome	MW430974
Epsilon	SARS-CoV-2/human/USA/CA-LACPHL-AF00014/2020, complete genome	MW411934
Delta	SARS-CoV-2/human/USA/IN-CDC-STM-000045992/2021, complete genome	MW931310
Omicron (BA.1)	SARS-CoV-2/human/BEL/rega-20174/2021	OL672836
Omicron (BA.2)	SARS-CoV-2/human/USA/FL-CDC-STM-77CPCCUR3/2022	OM371884
Omicron (BA.2.12.1)	SARS-CoV-2/human/USA/NY-CDC-LC0553978/2022	OM958567

Table S3. Major spike mutations of Alpha variants of concern found in the encoded proteins of SARS-CoV-2 strains

Time	H69del	V70del	Y144del	E484K	N501Y	D614G	P681H	T716I	S982A	D1118H
2021.01-2021.06	18.8% (6/32)	9.4% (3/32)	21.9% (7/32)	31.3% (10/32)	9.4% (3/32)	100% (32/32)	21.9% (7/32)	9.4% (3/32)	9.4% (3/32)	21.9% (7/32)
2021.07-2021.12	3.0% (1/33)	3.0% (1/33)	3.0% (1/33)	6.1% (2/33)	3.0% (1/33)	100% (33/33)	3.0% (1/33)	3.0% (1/33)	3.0% (1/33)	3.0% (1/33)
2022.01-2022.07	37.8% (14/37)	24.3% (9/37)	21.6% (8/37)	0% (0/37)	94.6% (35/37)	100% (37/37)	94.6% (35/37)	0% (0/37)	0% (0/37)	0% (0/37)

Table S4. Major spike mutations of Delta variants of concern found in the encoded proteins of SARS-CoV-2 strains

Time	T19R	T95I	G142D	E156del	F157del	R158G	A222V	K417N	L452R	T478K	D614G	P681R	D950N
2021.01-2021.06	6.3% (2/32)	0% (0/32)	6.3% (2/32)	6.3% (2/32)	6.3% (2/32)	6.3% (2/32)	6.3% (2/32)	0% (0/32)	12.5% (4/32)	6.3% (2/32)	100% (32/32)	6.3% (2/32)	6.3% (2/32)
2021.07-2021.12	90.9% (30/33)	0% (0/33)	90.9% (30/33)	90.9% (30/33)	90.9% (30/33)	90.9% (30/33)	63.6% (21/33)	0% (0/33)	90.9% (30/33)	90.9% (30/33)	100% (33/33)	90.9% (30/33)	87.9% (29/33)
2022.01-2022.07	2.7% (1/37)	21.6% (8/37)	94.6% (35/37)	2.7% (1/37)	2.7% (1/37)	2.7% (1/37)	2.7% (1/37)	45.9% (17/37)	21.6% (8/37)	97.3% (36/37)	100% (37/37)	2.7% (1/37)	2.7% (1/37)

Table S5. Major spike mutations of Epsilon variants of concern found in the encoded proteins of SARS-CoV-2 strains

Time	S13I	W152C	D614G
2021.01-2021.06	6.3% (2/32)	6.3% (2/32)	100% (32/32)
2021.07-2021.12	0% (0/33)	0% (0/33)	100% (33/33)
2022.01-2022.07	0% (0/37)	0% (0/37)	100% (37/37)

Table S6. Major spike mutations of Omicron (BA.1.1) variants of concern found in the encoded proteins of SARS-CoV-2 strains

Omicron (BA.1.1)	A67V	H69del	V70del	T95I	V143del/Y144del/Y145del	N211del	L212I	S371L	G446S	G496S	T547K	N856K	L981F
2021.01-2021.06	0% (0/32)	18.8% (6/32)	9.4% (3/32)	0% (0/32)	21.9% (7/32)	0% (0/32)	0% (0/32)	0% (0/32)	0% (0/32)	0% (0/32)	0% (0/32)	0% (0/32)	0% (0/32)
2021.07-2021.12	0% (0/33)	3.0% (1/33)	3.0% (1/33)	0% (0/33)	3.0% (1/33)	0% (0/33)	0% (0/33)	0% (0/33)	0% (0/33)	0% (0/33)	0% (0/33)	0% (0/33)	0% (0/33)
2022.01-2022.07	21.6% (8/37)	37.8% (14/37)	24.3% (9/37)	21.6% (8/37)	21.6% (8/37)	18.9% (7/37)	18.9% (7/37)	21.6% (8/37)	21.6% (8/37)	21.6% (8/37)	21.6% (8/37)	21.6% (8/37)	21.6% (8/37)

Table S7. Major spike mutations of Omicron (BA.2) variants of concern found in the encoded proteins of SARS-CoV-2 strains

Omicron (BA.2)	T19I	L24del	A27S	V213G	S371F	T376A	D405N	R408S
2021.01-2021.06	0% (0/32)	0% (0/32)	0% (0/32)	0% (0/32)	0% (0/32)	0% (0/32)	0% (0/32)	0% (0/32)
2021.07-2021.12	0% (0/32)	0% (0/32)	0% (0/32)	0% (0/32)	0% (0/32)	0% (0/32)	0% (0/32)	0% (0/32)
2022.01-2022.07	70.3% (26/37)	73.0% (27/37)	73.0% (27/37)	75.7% (28/37)	70.3% (26/37)	70.3% (26/37)	70.3% (26/37)	70.3% (26/37)
